# Multimodal Magnetic Resonance Imaging Reveals Aberrant Brain Age Trajectory During Youth in Schizophrenia Patients

**DOI:** 10.3389/fnagi.2022.823502

**Published:** 2022-03-03

**Authors:** Jiayuan Huang, Pengfei Ke, Xiaoyi Chen, Shijia Li, Jing Zhou, Dongsheng Xiong, Yuanyuan Huang, Hehua Li, Yuping Ning, Xujun Duan, Xiaobo Li, Wensheng Zhang, Fengchun Wu, Kai Wu

**Affiliations:** ^1^Department of Biomedical Engineering, School of Material Science and Engineering, South China University of Technology, Guangzhou, China; ^2^School of Biomedical Sciences and Engineering, South China University of Technology, Guangzhou International Campus, Guangzhou, China; ^3^The Affiliated Brain Hospital of Guangzhou Medical University, Guangzhou Huiai Hospital, Guangzhou, China; ^4^Guangdong Engineering Technology Research Center for Translational Medicine of Mental Disorders, Guangzhou, China; ^5^MOE Key Lab for Neuroinformation, High-Field Magnetic Resonance Brain Imaging Key Laboratory of Sichuan Province, University of Electronic Science and Technology of China, Chengdu, China; ^6^Department of Biomedical Engineering, New Jersey Institute of Technology, Newark, NJ, United States; ^7^Institute of Automation, Chinese Academy of Sciences, Beijing, China; ^8^National Engineering Research Center for Tissue Restoration and Reconstruction, South China University of Technology, Guangzhou, China; ^9^Guangdong Province Key Laboratory of Biomedical Engineering, South China University of Technology, Guangzhou, China; ^10^Guangdong Engineering Technology Research Center for Diagnosis and Rehabilitation of Dementia, Guangzhou, China; ^11^Institute for Healthcare Artificial Intelligence Application, Guangdong Second Provincial General Hospital, Guangzhou, China; ^12^Department of Nuclear Medicine and Radiology, Institute of Development, Aging and Cancer, Tohoku University, Sendai, Japan

**Keywords:** schizophrenia, accelerated brain aging, brain age gap, multimodal magnetic resonance imaging, machine learning

## Abstract

Accelerated brain aging had been widely reported in patients with schizophrenia (SZ). However, brain aging trajectories in SZ patients have not been well-documented using three-modal magnetic resonance imaging (MRI) data. In this study, 138 schizophrenia patients and 205 normal controls aged 20–60 were included and multimodal MRI data were acquired for each individual, including structural MRI, resting state-functional MRI and diffusion tensor imaging. The brain age of each participant was estimated by features extracted from multimodal MRI data using linear multiple regression. The correlation between the brain age gap and chronological age in SZ patients was best fitted by a positive quadratic curve with a peak chronological age of 47.33 years. We used the peak to divide the subjects into a youth group and a middle age group. In the normal controls, brain age matched chronological age well for both the youth and middle age groups, but this was not the case for schizophrenia patients. More importantly, schizophrenia patients exhibited increased brain age in the youth group but not in the middle age group. In this study, we aimed to investigate brain aging trajectories in SZ patients using multimodal MRI data and revealed an aberrant brain age trajectory in young schizophrenia patients, providing new insights into the pathophysiological mechanisms of schizophrenia.

## Introduction

Schizophrenia (SZ) is one of the costliest mental disorders in terms of human suffering and societal expenditure, with a 1% lifetime risk, chronicity, severity, and an impaired quality of life (van Os and Kapur, 2009; Cocchi et al., 2011; Charlson et al., 2018). Regretfully, etiology of the disease is unknown. Recent studies have found structural abnormalities in SZ patients, including decreased fractional anisotropy, gray matter volume (GMV) and hippocampal volume (Ellison-Wright and Bullmore, 2009; Bois et al., 2016; Wu et al., 2018; Duan et al., 2021), but brain volume changes are not constant throughout the course of the illness (van Haren et al., 2008). Functional magnetic resonance imaging (MRI) studies have shown similar abnormalities in the brains of SZ patients, such as a decrease in the amplitude of low-frequency fluctuations (Huang et al., 2010), an increase in functional connectivity within the default mode network (He et al., 2013), and changes in network homogeneity (Guo et al., 2014). Importantly, structural and functional abnormalities result in different brain aging trajectories (Mitelman et al., 2009; Mandl et al., 2010). Several recent studies have revealed that some of the changes observed in SZ patients are similar to those seen in physiological aging (Douaud et al., 2009; Nenadic et al., 2012).

During the normal aging process, brain changes include highly coordinated and sequenced events characterized by both progressive (myelination) and regressive (synaptic pruning) processes (Silk and Wood, 2011). Healthy brain aging demonstrates a specific pattern, in which cortical GMV decreases curvilinearly, cortical white matter volume (WMV) remains constant, and cortical cerebrospinal fluid increases (Pfefferbaum et al., 1994). Therefore, brain age (BA) could be estimated by analyzing brain structure, function, and connectivity features over time (Dosenbach et al., 2010). Recently, machine learning methods for BA estimation modeling were introduced, in which individual neuroimage features were used for model training (Huang et al., 2017; Lewis et al., 2018; Bashyam et al., 2020), and the model can provide potential brain aging biomarkers (Kondo et al., 2015; Qin et al., 2015; Valizadeh et al., 2017).

Individuals with the same chronological age (CA) might experience different trajectories of brain aging. Such differences might result in a mismatch between the CA and the BA. Previous studies have shown that this mismatch occurs simultaneously with brain changes (Cole et al., 2015, 2018a). Importantly, this kind of mismatch has been observed in neuropsychiatric disorders (Koutsouleris et al., 2014; Nenadic et al., 2017; Kaufmann et al., 2019; He et al., 2020), and the degree of deviation from the CA might help in the detection of clinical outcomes (Franke et al., 2010; Wang et al., 2019). Cole et al. found that the brain age gap (BAG) between the BA and the CA of elderly individuals was associated with a higher risk of mental or physical problems, as well as premature death (Cole et al., 2018b,^2019^). Boyle et al. revealed that the BAG was related to specific cognitive functions (Boyle et al., 2021). However, the BA trajectory in SZ patients based on three-modal MRI data has not been well-documented.

In this study, we used the Brainnetome Atlas (BNA)^1^ (Fan et al., 2016) and the white matter parcellation map (WMPM) (Mori et al., 2008) to build feature vectors from multimodal MRI data, including structural MRI (sMRI), resting state-functional MRI (rs-fMRI) and diffusion tensor imaging (DTI) data. The value of the BAG was computed and provided an indication of deviation from normal aging trajectories. We estimated the BA and assessed specific patterns of brain aging trajectories in both the SZ and normal control (NC) groups. Based on previous evidence (van Haren et al., 2008; Mitelman et al., 2009; Mandl et al., 2010; Nenadic et al., 2012), we hypothesized that brain age in SZ patients might be increased and that the degree of brain aging might differ between age groups.

## Materials and Methods

### Participants

In this study, we recruited 363 subjects, including 154 SZ patients and 209 NCs, and collected their sMRI, rs-fMRI, and DTI data. SZ patients met the diagnostic criteria for the fourth edition of the Diagnostic and Statistical Manual of Mental Disorders (DSM-IV). SZ patients were recruited from the Affiliated Brain Hospital of Guangzhou Medical University, Guangzhou. All subjects were fully informed of the details of the experiment. They signed an informed consent before undergoing the clinical trial and MRI. This study was carried out in compliance with the Declaration of Helsinki and approved by the Ethics Committee of the Affiliated Brain Hospital of Guangzhou Medical University.

### Magnetic Resonance Imaging Acquisition

MRI data were acquired using a Philips 3T MR system (Philips, Achieva, Netherlands) located at the Affiliated Brain Hospital of Guangzhou Medical University. To ensure data quality, all MRI data were scanned using scanning protocols designed by experienced experts, and the instrument was operated by an imaging technologist. The participants were instructed to keep their eyes closed, relax but not sleep, and move as little as possible. For each subject, rs-fMRI data were collected using an echo-planar imaging (EPI) sequence (64*64 scan matrix with 3.4*3.4*4 mm^3^ spatial resolution, repetition time = 2,000 ms, echo time = 30 ms, field of view = 220*220 mm^2^, flip angle = 90°, number of layers = 36, and layer thickness = 4 mm, total time = 486 s). The sMRI data were obtained using a sagittal three-dimensional gradient-echo T1-weighted sequence (256*256*188 matrix with 1*1*1 mm^3^ spatial resolution, repetition time = 8.2 ms, echo time = 3.7 ms, flip angle = 7°, and layer thickness = 1 mm). The DTI data were acquired by applying a single-shot EPI-based sequence (spatial resolution = 2*2*3 mm^3^, field of view = 256*256 mm^2^, repetition time = 6,000 ms, echo time = 70 ms, flip angle = 90°, number of layers = 50, and layer thickness = 3 mm, 33 nonlinear diffusion weighting directions with b = 1,000 s/mm^2^, and one direction without diffusion weighting).

### Image Preprocessing

The preprocessing steps of multimodal MRI data were same to those in our previous studies (Wu et al., 2018; Kong et al., 2021; Zang et al., 2021). More details were described in the Supplementary Material. After the correction of head motion, 20 subjects (16 SZ patients and 4 NCs) with excessive head motion (2 mm or 2°) were excluded. Therefore, 138 SZ patients and 205 NCs were included in the analysis (Table 1).

**TABLE 1:** Participant demographics.

	NC group	SZ group	*p* value
Gender	110/95	95/43	<0.05^a^
Education years	12.84 ± 2.83	10.74 ± 3.29	<0.05^b^
Age (years)	32.51 ± 8.37	33.75 ± 7.23	0.15^b^
PANSS positive symptom scale score	–	23.26 ± 5.16	–
PANSS negative symptom scale score	–	22.60 ± 7.47	–
PANSS general psychopathology scale score	–	40.03 ± 9.55	–

*PANSS, positive and negative syndrome scale.*

*^a^p value was obtained by a chi-square test.*

*^b^p value was obtained by a two-sample t-test.*

#### Structural Magnetic Resonance Imaging

The SPM12^2^ packages were used to preprocess the sMRI data. The whole brain was parcellated by the BNA containing 210 cortical and 36 subcortical regions. The GMV and WMV values of 246 brain regions were calculated.

#### Resting-State Functional Magnetic Resonance Imaging

The SPM12 and DPARSF^3^ packages (Yan and Zang, 2010) were used to preprocess the rs-fMRI data. The amplitude of low frequency fluctuation (ALFF), regional homogeneity (ReHo), and degree centrality (DC) values of 246 brain regions were computed.

#### Diffusion Tensor Imaging

Diffusion tensor imaging data were preprocessed using the PANDA toolbox (Cui et al., 2013). The values of fractional anisotropy (FA), mean diffusivity (MD), axial diffusivity (AD), and radial diffusivity (RD) of 50 white matter regions defined by the WMPM were calculated.

### Age Estimation Analysis

#### Feature Selection

In the current study, 2 sMRI indices (i.e., GMV and WMV), 3 rs-fMRI indices (i.e., ALFF, ReHo, and DC) of 246 brain regions, as well as 4 DTI indices (i.e., FA, MD, AD and RD) of 50 brain regions were extracted for each subject. As a result, a feature vector with 1,430 dimensions was obtained for each subject. The vectors were normalized by the following formula:


Z=(x-u)/s

where *u* and *s* are the mean and standard deviation of the training subjects’ features, respectively.

The model is likely to overfit when the number of features is much larger than the number of samples (Erickson et al., 2017). Thus, the feature selection procedure might be useful and critical for improving prediction accuracy. To obtain the age-related features, least absolute shrinkage and selection operator with cross-validation (LASSOCV) (Tibshirani, 1996) was performed. The LASSOCV sets most features to zeros and retains correlated ones by using L1-norm, which results in a very sparse features matrix and reduce dimensions of features.

#### Brain Age Estimation

**FIGURE 1:**
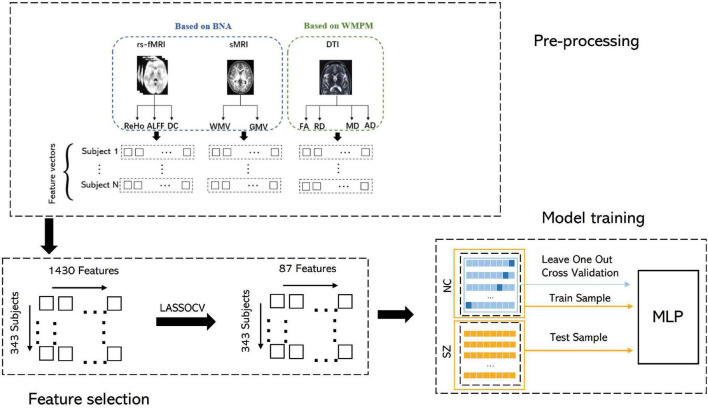
Methodological sketch.

Multiple linear regression (MLR) is a standard statistical technique for predicting the criterion or the independent variable by linearly combining several variables. MLR does not have algorithm-specific parameters. The measure of MLR is the mean absolute error (MAE), Pearson correlation, coefficient of determination and root mean squared error (rMSE). Notably, MLR has been successfully used in neuroimaging studies to assess age (Valizadeh et al., 2017) and other scores (Rosenberg et al., 2016; Shen et al., 2017). BA was estimated in NCs using leave-one-out cross-validation (LOOCV) (Figure 1). Furthermore, to investigate MLR prediction accuracy with different modal features, we repeated the above processes using different modal features. Data from SZ patients were used for testing based on the model of MLR trained by all NC subjects. The BA numeric value may indicate the degree of brain aging. The mean BAG of the NC group should consequently be or close to zero.

#### Statistical Analysis

Two-sample *t*-tests were used for the statistical analysis of between-group differences in age and education years. A chi-square test was used for the statistical analysis of gender differences. We employed linear regression to remove the effects of gender on features (Kondo et al., 2015). A permutation test was applied to estimate the statistical significance of the MAE results like classification (Golland and Fischl, 2003). In our analysis, we randomly permuted the age labels of the training data 1,000 times and performed all regression processes with each set of permuted labels. Quadratic and linear models were used to fit the BAG and the CA in the SZ group, with the positive quadratic model performing better (Supplementary Figure 1), similar result was acquired by BA estimation based on sMRI and DTI (Supplementary Figure 3). Thus, the CA of the peak of the positive quadratic curve was used to divide the subjects into a youth group and a middle age group. The Pearson correlation analysis between BA and CA in NC group was used to estimate model prediction accuracy, while correlation analyses between BAG and CA in SZ group were used to identify the relation between BAG and CA. We defined subjects with age less than the peak of the quadratic curve as the youth group, others as the middle age group. Because the brain structure and function of SZ patients differed from those of NCs (Mori et al., 2007; Bose et al., 2009), differences in the BA and the CA were investigated by two-sample *t*-tests in the SZ and NC groups, the BAG between the SZ and NC groups was compared in the youth and middle age groups. Prior to statistical analyses, the BA was corrected with the following formula (de Lange and Cole, 2020):


o⁢f⁢f⁢s⁢e⁢t=α×C⁢A+β

c⁢o⁢r⁢r⁢e⁢c⁢t⁢e⁢d⁢B⁢A=B⁢A-o⁢f⁢f⁢s⁢e⁢t

Where the offset = BA-CA, the coefficient α and β represent the slope and intercept.

## Results

### Characterization of Age-Related Features

**FIGURE 2:**
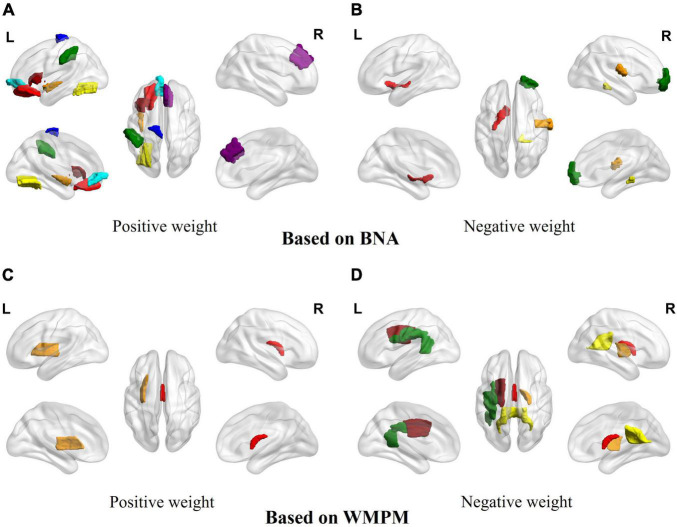
The signed importance of brain regions for BA predictions in the MLP model. **(A)** Brain areas with positive weights based on the BNA. Red: orbital gyrus/frontal lobe; brown: insular gyrus/insula lobe; yellow: fusiform gyrus/temporal lobe; green: parietal lobule/inferior parietal lobe; dark red: inferior frontal gyrus/frontal lobe; cyan: orbital gyrus/frontal lobe; blue: precentral gyrus/frontal lobe; purple: superior frontal gyrus/frontal lobe. **(B)** Brain areas with negative weights based on the BNA. Red: striatum/subcortical nuclei; brown: postcentral gyrus/parietal lobe; yellow: parahippocampal gyrus/temporal lobe; green: middle temporal gyrus/temporal lobe. **(C)** Brain areas with positive weights based on the WMPM. Red: fornix; brown: external capsule. **(D)** Brain areas with negative weights based on the WMPM. Red: fornix; brown: posterior limb of internal capsule; yellow: splenium of corpus callosum; green: superior longitudinal fasciculus; dark red: superior corona radiata.

Eighty-seven of the 1,430 features were more relevant to age and were used for the BA estimation in the NC group after LASSOCV (alpha = 0.3). The sMRI, rs-fMRI, and DTI data accounted respectively for 50, 24, and 13 features in the final feature set, respectively. After ranking the weights of all features in the MLR, the 20 best descriptors were as follows: the values of 11 brain regions in the BNA (the orbital gyrus, fusiform gyrus, striatum, precentral gyrus, superior frontal gyrus, postcentral gyrus, parahippocampal gyrus, middle temporal gyrus, insular gyrus, inferior parietal lobule and inferior frontal gyrus); and the values of 6 brain regions in the WMPM (fornix, posterior limb of internal capsule, superior corona radiata, superior longitudinal fasciculus, splenium of corpus callosum and external capsule) (see Table 2 and Figure 2).

**TABLE 2:** Brain regions with high weights in BA estimation.

Feature	Weight	Atlas	Region
MD	−6.517	WMPM	Fornix (column and body of fornix)
FA	−4.094	WMPM	Fornix (column and body of fornix)
GMV	−1.522	BNA	Subcortical nuclei/Striatum (L)
MD	−1.448	WMPM	Posterior limb of internal capsule (R)
GMV	−1.398	BNA	Parietal lobe/Postcentral gyrus (R)
ReHo	−1.193	BNA	Temporal lobe/Parahippocampal gyrus (R)
FA	−1.115	WMPM	Splenium of corpus callosum
FA	−1.091	WMPM	Superior longitudinal fasciculus (L)
ReHo	−1.055	BNA	Temporal lobe/Middle temporal gyrus (R)
FA	−0.889	WMPM	Superior corona radiata (L)
AD	3.857	WMPM	Fornix (column and body of fornix)
WMV	1.133	BNA	Frontal lobe/Orbital gyrus (L)
ALFF	0.922	BNA	Insular lobe/Insular gyrus (L)
WMV	0.849	BNA	Temporal lobe/Fusiform gyrus (L)
DC	0.845	BNA	Parietal lobe/Inferior parietal lobule (L)
WMV	0.838	BNA	Frontal lobe/Inferior frontal gyrus (L)
MD	0.821	WMPM	External capsule (L)
WMV	0.705	BNA	Frontal lobe/Orbital gyrus (L)
WMV	0.705	BNA	Frontal lobe/Precentral gyrus (L)
WMV	0.635	BNA	Frontal lobe/Superior frontal gyrus (R)

*L is left and R is right.*

### Correlation Between Brain Age and Chronological Age in the Youth and Middle Age Groups in Normal Controls and Schizophrenia Patients

**FIGURE 3:**
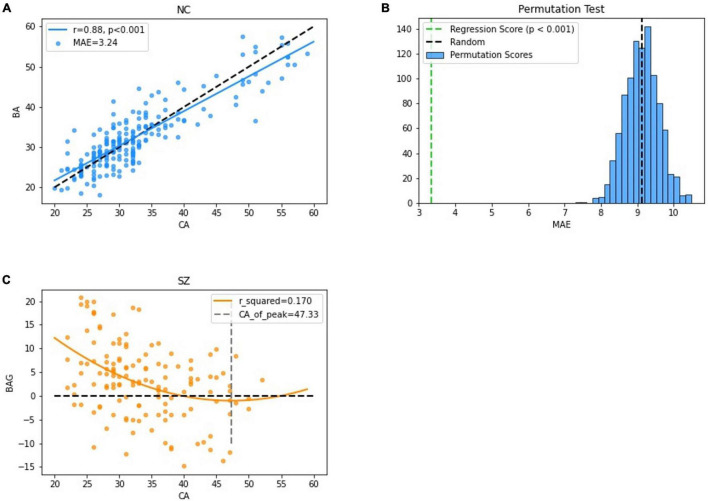
Performance of the BA estimation model. **(A)** The correlation between the CA and the BA in the NC group. **(B)** The results of permutation tests of the BA estimation model. **(C)** The correlation between the BAG and the CA in the SZ group.

**FIGURE 4:**
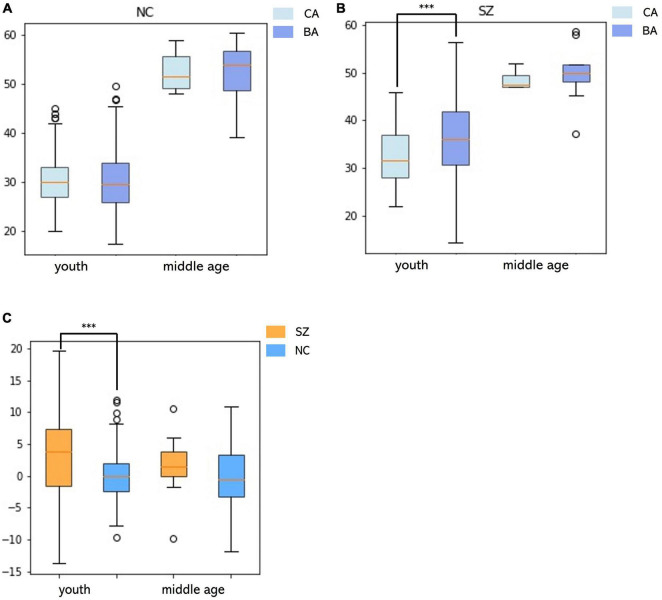
Relationships between the BA and the CA and differences in the BAG in youth and middle age for the SZ and NC groups. **(A)** In the NC group, differences between BA and CA in the youth and middle age groups. **(B)** In the SZ group, differences between BA and CA in the youth and middle age groups. **(C)** Differences in BAG between SZ and NC groups in youth and middle age.

The MLR in the NC group had the best accuracy based on different model MRI combinations (Supplementary Table 1), in which the Pearson correlation, MAE, coefficient of determination and rMSE of the MLR were 0.88, 3.24 years, 0.77 and 4.14 years respectively (uncorrected) and significant in the permutation test. In the SZ group, the best model used to fit the BAG and CA was the positive quadratic regression, in which the peak CA was 47.33 years (Figure 3 and Supplementary Figure 1). The peak was then used to divide the subjects into the youth (age range: 22–46 years, NC: 183, SZ: 128) and middle age (age range: 47–60 years, NC: 22, SZ: 10) groups. Before two-sample *t*-tests performed, BA was corrected (see in Supplementary Figure 2 and Supplementary Table 3). In the youth SZ group, the BA values were significantly higher than the CA values. In the middle age SZ group, the BA and CA values were not significantly different. When the same analysis was performed on the NC group, no significant differences were found in the youth group or in the middle age group. Accordingly, the BAG of the youth SZ group was significantly higher than that of the youth NC group. However, the BAG of the middle age SZ group was also higher than that of the middle age NC group, despite the fact that the difference was not statistically significant (Figure 4). The results were similar in BA estimation based on sMRI and DTI (Supplementary Figure 4). This finding might be associated with the atypical brain structure and function observed in SZ patients.

## Discussion

To the best of our knowledge, this was the first study to use three-modal MRI data to analyze brain aging trajectories in SZ patients. A positive quadratic trajectory of the BAG with the CA was found in the SZ group. SZ patients showed significantly higher BA than CA in the youth group, implying that youth SZ patients have increased brain age.

### Methodology Consideration

Recent studies have predicted the BA based on unimodal neuroimaging and biological information, but few have combined three-modal MRI features (Dosenbach et al., 2010; Brown et al., 2012; Lewis et al., 2018). In the current study, we combined sMRI, rs-fMRI and DTI to estimate the BA. Combined multimodal MRI features are of crucial importance because they can identify different patterns of alterations in the microstructure and macrostructure of the brain (Cherubini et al., 2016). de Lange et al. showed that combining features from multimodal MRI data (T1-weighted MRI, DTI, and rs-fMRI) yielded better age prediction performance than using unimodal features (de Lange et al., 2020). The combination of T1-weighted and T2-weighted image features also yielded better prediction performance than unimodal features (Rokicki et al., 2021). Similarly, our results showed that combining multimodal MRI data can achieve sensitive detection of aberrant patterns in brain aging (Supplementary Table 1).

In addition, the LASSOCV was applied to selected features which were more relevant to age (Zhang and Huang, 2008). The MLR model allowed for fast computation and straightforward interpretation of feature weights. Due to number of subjects limitation, LOOCV was used to estimate model prediction accuracy.

### Brain Age Trajectories in Youth Schizophrenia Patients

In the SZ group, we found that the correlation between the BAG and the CA was best fitted by a positive quadratic curve. On the left side of the positive quadratic curve, the BAG decreased with CA. We speculated that the BA and CA trajectories became more convergent in young SZ patients. This result was consistent with previous findings of age-related abnormalities in the brain, showing that the brains of SZ patients undergo a recovery process to realign the brain to that of a NC (Bose et al., 2009; Voineskos et al., 2010). Moreover, Schnack et al. found that the acceleration of brain aging in SZ patients decreased from 2.5 years/year to a normal rate approximately 5 years after illness onset, which could be attributed to the medication effect (Schnack et al., 2016). Recent studies have shown that the medication effect has a nonlinear relationship with antipsychotic treatment dosage (Rhindress et al., 2017) and is related to the mean dynamic network interaction index of SZ patients (Wang et al., 2021). White matter trajectories are also changed in SZ patients. Disturbances during maturation would be reflected by different ascending slopes and a shift in peak white matter maturation (Cetin-Karayumak et al., 2020), implying that the white matter trajectories of SZ patients and NCs intersect (Bose et al., 2009; Wright et al., 2014; de Moura et al., 2018). Our results were consistent with previous findings that showed changing difference between the CA and the BA in SZ patients and further suggested that such abnormalities might differ at different ages. Therefore, we divided the participants into youth and middle age groups using the peak CA and found a trajectory of increased brain age in youth SZ patients. Specifically, the BA was significantly higher than the CA in youth SZ patients, whereas the BA and CA showed no significant difference in the middle age SZ patients. Similarly, Voineskos et al. found that young SZ patients had a significantly lower FA than young NCs, but no differences were found when the older groups were compared (Voineskos et al., 2010). van Haren et al. showed that excessive volume loss in SZ patients did not occur to the same degree throughout the course of the illness, and it was most prominent during the first two decades (van Haren et al., 2008).

### Important Features for Brain Age Estimation Based on the Brainnetome Atlas

Our results suggested that subcortical nuclei, the frontal lobe and the temporal lobe all play important roles in BA estimation. Subcortical structures are responsible for exerting cognitive, affective, and social functions in humans (Koshiyama et al., 2018). Our results showed that key subcortical structure was the striatum, which play important roles in decision-making (Goulet-Kennedy et al., 2016). Previous studies have indicated that aging causes significant changes in intrinsic functional connectivity in the striatum (Porter et al., 2015). Abnormalities in the striatum may be related to various neuropsychiatric disorders, including SZ, bipolar disorder, and attention deficit hyperactivity disorder (Emond et al., 2009; Karcher et al., 2019).

The frontal lobe is thought to manage incoming information and select appropriate actions based on one’s goals in a particular context (Rosch and Mostofsky, 2019). The orbital and precentral gyri in the frontal lobe were also important for BA estimation and which are associated with emotional expression and motor behaviors (Dixon et al., 2017; Zhou et al., 2020). Accumulating evidence suggests that the orbital and precentral gyri may be abnormal in neuropsychiatric diseases such as somatic depression, bipolar disorder, autism spectrum disorder and SZ (Stanfield et al., 2009; Nebel et al., 2014; Zarei, 2018; Yan et al., 2019).

The main functions of the temporal lobe are to process auditory information and encode of memory (Hamberger et al., 2003; Dickerson et al., 2004). The fusiform and parahippocampal gyri in the temporal lobe with high weight are responsible for processing visual information and memory (Luck et al., 2010; Weiner and Zilles, 2016). Fusiform abnormality has been linked to SZ (McKenna et al., 2019). Atrophy of the parahippocampal gyrus has also been implicated as an early indicator of Alzheimer’s disease (Echavarri et al., 2011).

### Important Features for Brain Age Estimation Based on the White Matter Parcellation Map

Different brain regions are linked by white matter fibers, and different white matter fibers are associated with various behaviors. The splenium of corpus callosum and external capsule, which are linking different brain regions, played major roles in BA estimation model. The functions of splenium of corpus callosum and external capsule are linking primary and secondary visual areas and serving as a route for cholinergic fibers from basal forebrain to the cerebral cortex (Knyazeva, 2013; Nolze-Charron et al., 2020), respectively. These fibers would change during the course of normal aging (Nolze-Charron et al., 2020; Delvenne et al., 2021). Abnormalities of the fibers were found in SZ (Francis et al., 2011; Joo et al., 2021).

The superior longitudinal fasciculus, posterior limb of the internal capsule, superior corona radiata and fornix, which are associated with the language, motor and memory (Jang, 2009; Bernal and Altman, 2010; Adnan et al., 2013), were all given high weight in the BA estimation model. A recent study found that some of these brain regions are age-related and showed lower FA values in SZ patients than in NCs (Yoshimura and Kurashige, 2000; Peters et al., 2010; Kochunov et al., 2016; Tesli et al., 2021). Accumulating evidence suggests that the brain regions are affected in psychiatric disorders, such as SZ, bipolar disorder and Alzheimer’s disease (Koshiyama et al., 2020; Linke et al., 2020).

### Contribution of Simultaneous Multimodal Magnetic Resonance Imaging Data

Our findings demonstrated that both some brain regions have critical contributions to BA estimation. The use of multimodal neuroimages results in a more sensitive model that captures alterations in brain structure and function (Cherubini et al., 2016). The results for the BAG in SZ youth group could explain altered brain functions such as decision-making as well as changes in visual, language, motor, memory, and emotional expression. Thus, future studies using multimodal neuroimaging are necessary for broadening our understanding of brain aging.

### Limitations

The findings of this study should be considered in light of some limitations. First, the sample size was relatively small, especially in the middle age group. The lack of significant results between NC and SZ in the middle age group could be explained by a small group sample size. We utilized the data of the NC group to train the model and discover age-related brain regions that provided useful information on normal aging patterns rather than an independent NC dataset. Future work should train predictive models using data from a large number of NCs and test the models using an independent dataset. Second, we did not consider factors such as the years of illness and medication. Third, a longitudinal study can be used to validate the aberrant trajectories in SZ patients.

## Conclusion

We found a positive quadratic trajectory between the BAG and the CA in SZ patients. BA was significantly higher than CA only in the youth SZ group but not significantly in the middle age group. These results suggested that discrepancies between BA and CA might be attributed to abnormal brain aging trajectories in SZ patients and demonstrated that SZ patients exhibit varying degrees of increased brain age at different age ranges. Furthermore, we suggest that the BA predicted by three-modal MRI data support more comprehensive biomarker for understanding abnormal brain aging patterns in SZ patients.

## Data Availability Statement

The datasets presented in this article are not readily available because this is a private dataset. Requests to access the datasets should be directed to KW, kaiwu@scut.edu.cn.

## Ethics Statement

The studies involving human participants were reviewed and approved by Affiliated Brain Hospital of Guangzhou Medical University. The patients/participants provided their written informed consent to participate in this study. Written informed consent was obtained from the individual(s) for the publication of any potentially identifiable images or data included in this article.

## Author Contributions

All authors contributed to data analysis, drafting and critically revising the manuscript, gave final approval of the version to be published, and agreed to be accountable for all aspects of the work.

## Conflict of Interest

The authors declare that the research was conducted in the absence of any commercial or financial relationships that could be construed as a potential conflict of interest.

## Publisher’s Note

All claims expressed in this article are solely those of the authors and do not necessarily represent those of their affiliated organizations, or those of the publisher, the editors and the reviewers. Any product that may be evaluated in this article, or claim that may be made by its manufacturer, is not guaranteed or endorsed by the publisher.

## Footnotes

1
http://atlas.brainnetome.org/

2
http://www.fil.ion.ucl.ac.uk/spm/

3
http://rfmri.org/DPARSF
