# Supplementary material for: Multimodal Magnetic Resonance Imaging Reveals Aberrant Brain Age Trajectory During Youth in Schizophrenia Patients
Source: Front Aging Neurosci. 2022 Mar 3;14:823502. doi: 10.3389/fnagi.2022.823502 (PMC8929292; doi:10.3389/fnagi.2022.823502)
Supplement: Supplementary file 1 [file Data_Sheet_1.DOCX]

**Images Preprocessing**

**Structure MRI**

We used VBM and SPM12 to preprocess structure MRI (sMRI) data of subjects. The steps are as follow: 1) adjustment of origin: to better align the images into the MNI standard space, the origin of all sMRI data was adjusted to the position of the former joint; 2) spatial normalization: the images were normalized to the MNI standard space using the high-dimensional DARTEL method; 3) segmentation: the images were segmented into gray matter and white matter after spatial normalization; 4) modulation: modulate the gray matter image after segmentation to eliminate the volume variation caused by individual differences in the subject's brain during spatial normalization; 5) smoothing: Smooth the segmented and modulated images using an 8mm Gaussian kernel.

**Resting-State Functional MRI**

SPM12 (<http://www.fil.ion.ucl.ac.uk/spm/>) and DPARSF (<http://rfmri.org/DPARSF>) were used to preprocessing resting-state-functional MRI (rs-fMRI) of subjects with following steps: 1) we removed the first 10 time points of the images due to possible signal instability caused by the magnetic field not reaching equilibrium; 2) time layer correction: since the MR scanner scans images layer by layer, the scan time of each layer is not consistent, and the time layer of the remaining images is corrected for the difference in scan time by using the middle layer as a reference; 3) head motion correction: to reduce the noise generated by head motion on the signal, 24 head motion parameters were estimated using the head motion correction algorithm; 4) remove the effect of global signals; 5) normalize the images: original spatial images of each subject were aligned to the standard space by affine transformation in order to reduce the differences between brain structures of different subjects, using the standard EPI template based on the SPM. The standard EPI template based on SPM was used to align all images to the MNI standard space, thus allowing human brains of different shapes and sizes to be compared with each other; 6) remove linear trends and some confounding signals: we removed the signals for reducing the effects of head movement and non-neuronal oscillatory signals; 7) finally, we used band-pass filtering (0.01-0.08 HZ) for each voxel time series for reducing the effects of high frequency physiological respiration, cardiac noise signals and low frequency drift noise.

**Diffusion Tensor Imaging**

DTI data were preprocessed using the PANDA toolbox. The specific steps are as follows: 1) skill stripping; 2) head movement and eddy current correction: eddy current was an important factor for image deformation, and affine transformation was used to register the DTI image to the T1 image, which can effectively reduce the influence of head movement and eddy current; 3) gradient orientation correction: head movement in the previous step is performed correction, the gradient direction correction is performed for each image at the same time.
