# Supplementary figures and images for: Multimodal Magnetic Resonance Imaging Reveals Aberrant Brain Age Trajectory During Youth in Schizophrenia Patients
Source: Front Aging Neurosci. 2022 Mar 3;14:823502. doi: 10.3389/fnagi.2022.823502 (PMC8929292; doi:10.3389/fnagi.2022.823502)

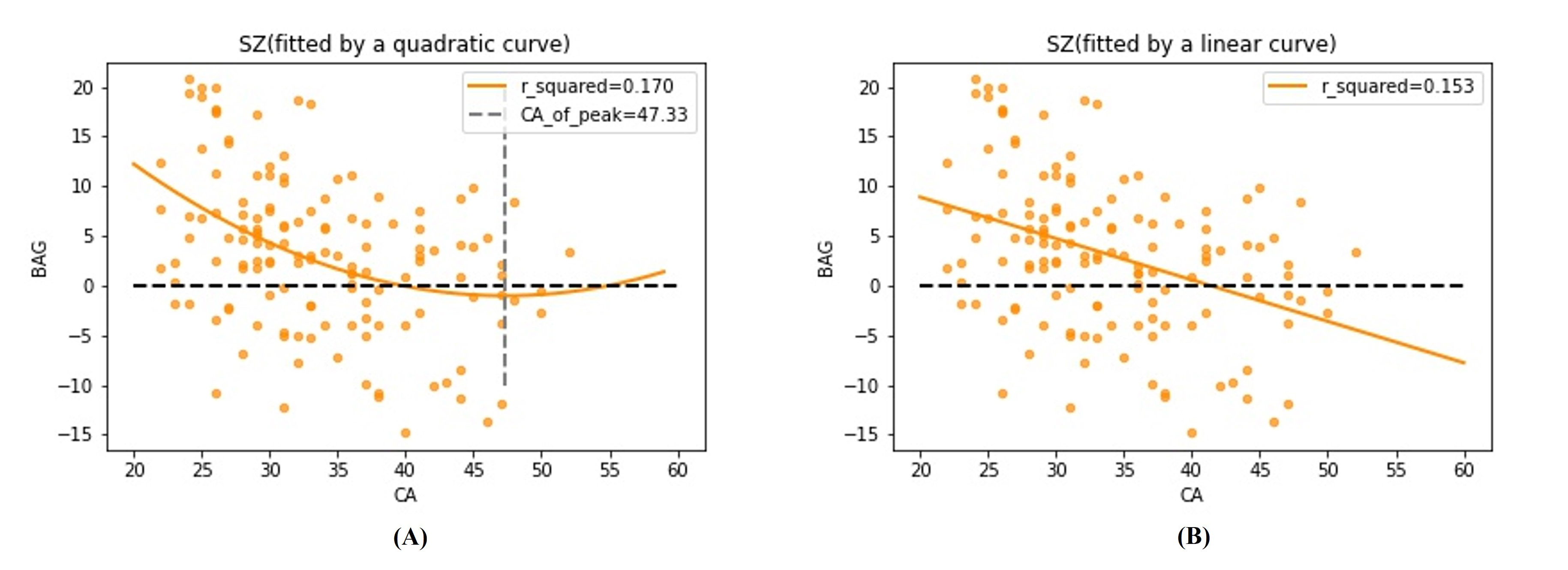

Supplement: Supplementary file 2 [file Image_1.JPEG]

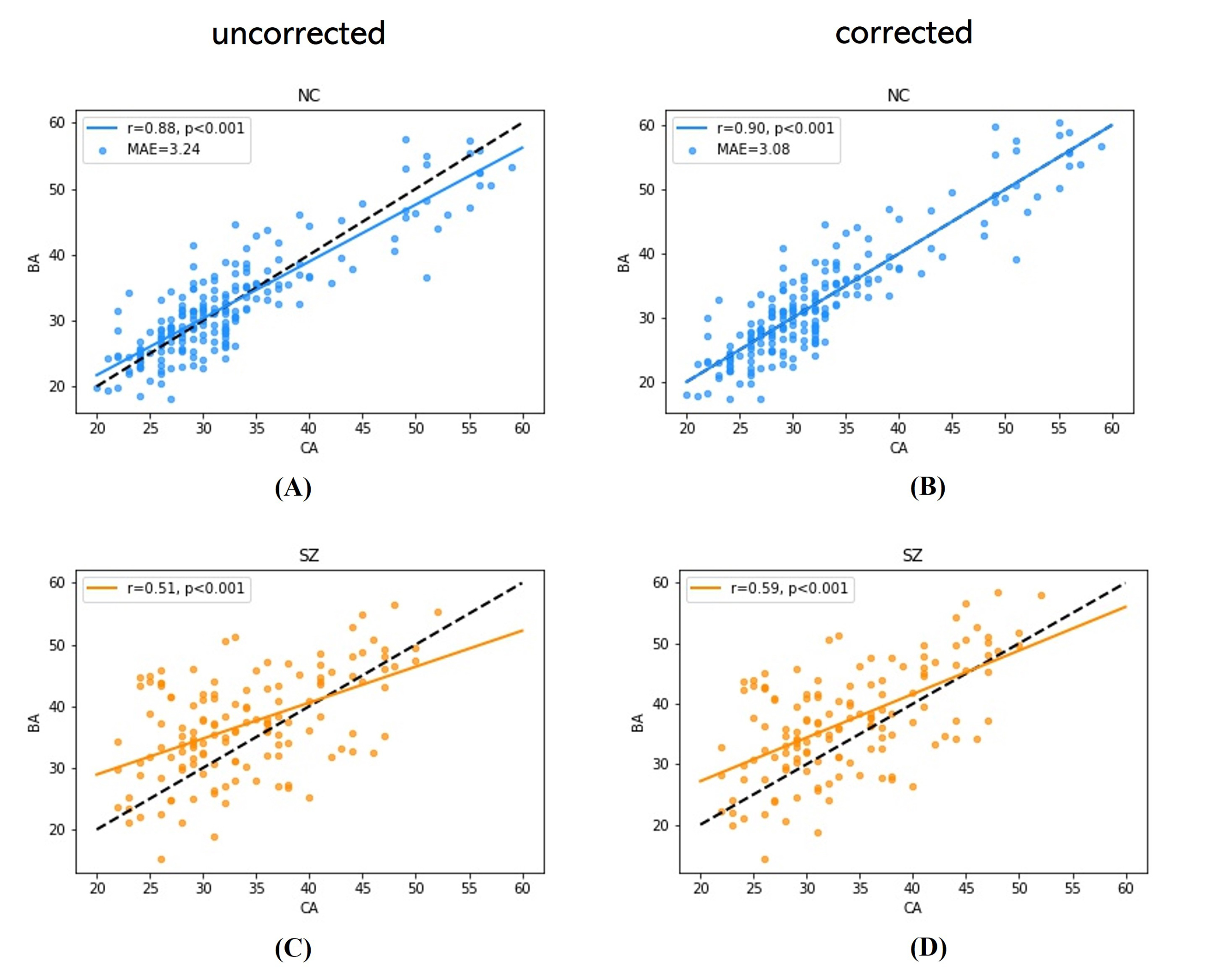

Supplement: Supplementary file 3 [file Image_2.JPEG]

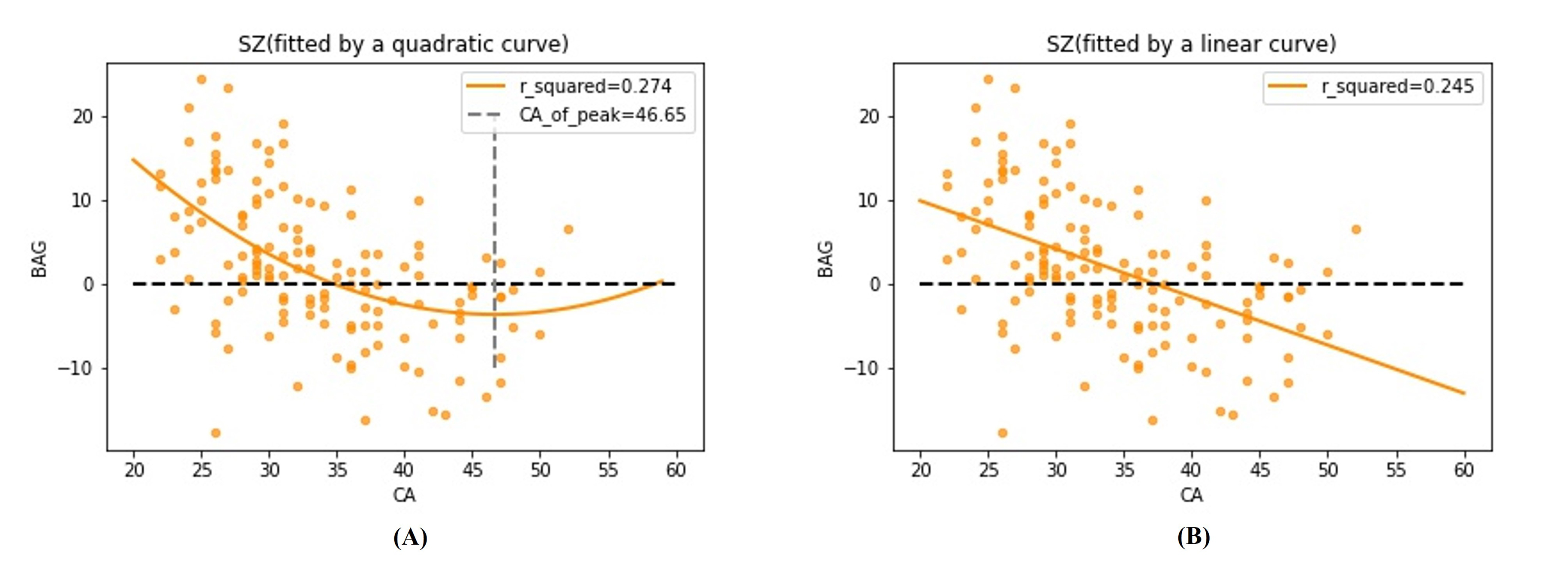

Supplement: Supplementary file 4 [file Image_3.JPEG]

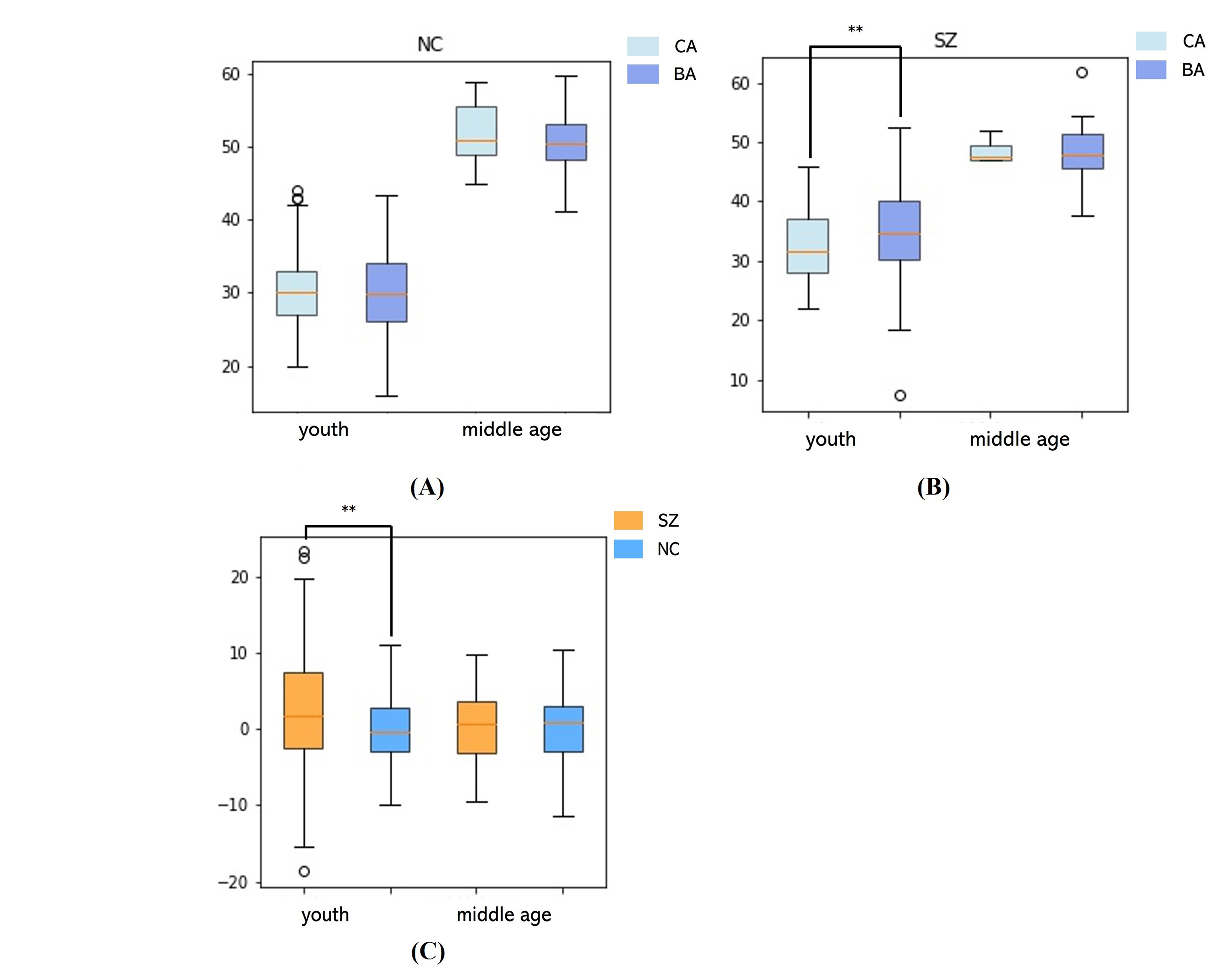

Supplement: Supplementary file 5 [file Image_4.JPEG]
