# Supplementary material for: Multimodal Magnetic Resonance Imaging Reveals Aberrant Brain Age Trajectory During Youth in Schizophrenia Patients
Source: Front Aging Neurosci. 2022 Mar 3;14:823502. doi: 10.3389/fnagi.2022.823502 (PMC8929292; doi:10.3389/fnagi.2022.823502)
Supplement: Supplementary file 6 [file Table_1.DOCX]

Table S1 Performance of BA (uncorrected) estimation model based on different modal neuroimaging data combination

| Modality | r | MAE | Coefficient of determination | rMSE |
| --- | --- | --- | --- | --- |
| sMRI | 0.79 | 3.99 | 0.62 | 5.23 |
| rs-fMRI | 0.70 | 4.88 | 0.49 | 6.09 |
| DTI | 0.72 | 4.74 | 0.52 | 5.93 |
| sMRI + DTI | 0.86 | 3.39 | 0.75 | 4.26 |
| sMRI + rs-fMRI | 0.78 | 4.13 | 0.62 | 5.33 |
| rs-fMRI + DTI | 0.81 | 3.85 | 0.67 | 4.97 |
| sMRI + rs-fMRI + DTI | 0.88 | 3.24 | 0.77 | 4.14 |
