# Supplementary material for: Multimodal Magnetic Resonance Imaging Reveals Aberrant Brain Age Trajectory During Youth in Schizophrenia Patients
Source: Front Aging Neurosci. 2022 Mar 3;14:823502. doi: 10.3389/fnagi.2022.823502 (PMC8929292; doi:10.3389/fnagi.2022.823502)
Supplement: Supplementary file 7 [file Table_2.DOCX]

Table S2 Weight of brain regions in MLR

| Feature | Weight | Atlas | Region |
| --- | --- | --- | --- |
| MD | -6.517 | WMPM | Fornix (column and body of fornix) |
| FA | -4.094 | WMPM | Fornix (column and body of fornix) |
| GMV | -1.522 | BNA | Subcortical Nuclei/Striatum (L) |
| MD | -1.448 | WMPM | Posterior limb of internal capsule (R) |
| GMV | -1.398 | BNA | Parietal Lobe/Postcentral Gyrus (R) |
| ReHo | -1.193 | BNA | Temporal Lobe/Parahippocampal Gyrus (R) |
| FA | -1.115 | WMPM | Splenium of corpus callosum |
| FA | -1.091 | WMPM | Superior longitudinal fasciculus (L) |
| ReHo | -1.055 | BNA | Frontal Lobe/Middle Frontal Gyrus (R) |
| FA | -0.889 | WMPM | Superior corona radiata (L) |
| ReHo | -0.703 | BNA | Limbic Lobe/Cingulate Gyrus (R) |
| WMV | -0.679 | BNA | Insular Lobe/Insular Gyrus (L) |
| ReHo | -0.673 | BNA | Temporal Lobe/Parahippocampal Gyrus (R) |
| WMV | -0.652 | BNA | Subcortical Nuclei/Thalamus (R) |
| GMV | -0.650 | BNA | Frontal Lobe/Inferior Frontal Gyrus (R) |
| WMV | -0.635 | BNA | Temporal Lobe/Posterior Superior Temporal Sulcus (L) |
| RD | -0.632 | WMPM | Superior cerebellar peduncle (R) |
| ReHo | -0.620 | BNA | Temporal Lobe/Middle Temporal Gyrus (L) |
| RD | -0.620 | WMPM | Body of corpus callosum |
| ReHo | -0.611 | BNA | Subcortical Nuclei/Amygdala (L) |
| WMV | -0.594 | BNA | Parietal Lobe/Precuneus (R) |
| ReHo | -0.578 | BNA | Temporal Lobe/Superior Temporal Gyrus (L) |
| FA | -0.562 | WMPM | Posterior limb of internal capsule (L) |
| GMV | -0.553 | BNA | Frontal Lobe/Middle Frontal Gyrus (L) |
| WMV | -0.542 | BNA | Insular Lobe/Insular Gyrus (R) |
| ReHo | -0.499 | BNA | Temporal Lobe/Inferior Temporal Gyrus (L) |
| ReHo | -0.490 | BNA | Occipital Lobe/Cuneus (L) |
| GMV | -0.472 | BNA | Limbic Lobe/Cingulate Gyrus (L) |
| ReHo | -0.327 | BNA | Frontal Lobe/Orbital Gyrus (R) |
| WMV | -0.320 | BNA | Temporal Lobe/Inferior Temporal Gyrus (R) |
| ReHo | -0.303 | BNA | Subcortical Nuclei/Amygdala (L) |
| WMV | -0.292 | BNA | Temporal Lobe/Posterior Superior Temporal Sulcus (L) |
| GMV | -0.275 | BNA | Temporal Lobe/Inferior Temporal Gyrus (L) |
| GMV | -0.270 | BNA | Subcortical Nuclei/Striatum (L) |
| FA | -0.197 | WMPM | Inferior cerebellar peduncle (R) |
| ReHo | -0.192 | BNA | Occipital Lobe/Occipital Gyrus (L) |
| DC | -0.172 | BNA | Subcortical Nuclei/Striatum (R) |
| WMV | -0.154 | BNA | Insular Lobe/Insular Gyrus (L) |
| WMV | -0.147 | BNA | Frontal Lobe/Middle Frontal Gyrus (L) |
| ALFF | -0.127 | BNA | Temporal Lobe/Superior Temporal Gyrus (R) |
| WMV | -0.088 | BNA | Subcortical Nuclei/Thalamus (R) |
| ReHo | -0.051 | BNA | Temporal Lobe/Superior Temporal Gyrus (L) |
| DC | -0.022 | BNA | Insular Lobe/Insular Gyrus (R) |
| WMV | -0.014 | BNA | Frontal Lobe/Middle Frontal Gyrus (R) |
| GMV | -0.006 | BNA | Subcortical Nuclei/Thalamus (L) |
| WMV | 0.006 | BNA | Parietal Lobe/Superior Parietal Lobule (R) |
| DC | 0.035 | BNA | Frontal Lobe/Orbital Gyrus (R) |
| WMV | 0.063 | BNA | Frontal Lobe/Superior Frontal Gyrus (L) |
| WMV | 0.082 | BNA | Parietal Lobe/Inferior Parietal Lobule (L) |
| WMV | 0.131 | BNA | Limbic Lobe/Cingulate Gyrus (L) |
| RD | 0.135 | WMPM | Anterior limb of internal capsule (R) |
| WMV | 0.196 | BNA | Parietal Lobe/Postcentral Gyrus (R) |
| WMV | 0.233 | BNA | Occipital Lobe/Occipital Gyrus (R) |
| WMV | 0.234 | BNA | Temporal Lobe/Middle Temporal Gyrus (L) |
| WMV | 0.239 | BNA | Parietal Lobe/Postcentral Gyrus (R) |
| WMV | 0.254 | BNA | Frontal Lobe/Middle Frontal Gyrus (L) |
| ReHo | 0.268 | BNA | Frontal Lobe/Superior Frontal Gyrus (L) |
| WMV | 0.270 | BNA | Parietal Lobe/Postcentral Gyrus (L) |
| WMV | 0.283 | BNA | Frontal Lobe/Orbital Gyrus (R) |
| WMV | 0.294 | BNA | Occipital Lobe/Cuneus (L) |
| WMV | 0.310 | BNA | Limbic Lobe/Cingulate Gyrus (L) |
| WMV | 0.317 | BNA | Frontal Lobe/Precentral Gyrus (R) |
| WMV | 0.329 | BNA | Frontal Lobe/Superior Frontal Gyrus (R) |
| GMV | 0.348 | BNA | Temporal Lobe/Parahippocampal Gyrus (L) |
| WMV | 0.358 | BNA | Parietal Lobe/Superior Parietal Lobule (L) |
| DC | 0.366 | BNA | Subcortical Nuclei/Thalamus (R) |
| GMV | 0.373 | BNA | Temporal Lobe/Parahippocampal Gyrus (R) |
| GMV | 0.373 | BNA | Temporal Lobe/Parahippocampal Gyrus (L) |
| WMV | 0.402 | BNA | Parietal Lobe/Precuneus (R) |
| DC | 0.406 | BNA | Parietal Lobe/Precuneus (R) |
| ReHo | 0.411 | BNA | Subcortical Nuclei/Thalamus (L) |
| WMV | 0.469 | BNA | Temporal Lobe/Parahippocampal Gyrus (L) |
| ReHo | 0.499 | BNA | Temporal Lobe/Inferior Temporal Gyrus (L) |
| WMV | 0.536 | BNA | Subcortical Nuclei/Striatum (R) |
| WMV | 0.563 | BNA | Frontal Lobe/Orbital Gyrus (R) |
| WMV | 0.568 | BNA | Temporal Lobe/Fusiform Gyrus (L) |
| WMV | 0.615 | BNA | Frontal Lobe/Inferior Frontal Gyrus (L) |
| WMV | 0.635 | BNA | Frontal Lobe/Superior Frontal Gyrus (R) |
| WMV | 0.705 | BNA | Frontal Lobe/Precentral Gyrus (L) |
| WMV | 0.705 | BNA | Frontal Lobe/Orbital Gyrus (L) |
| MD | 0.821 | WMPM | External capsule (L) |
| WMV | 0.839 | BNA | Frontal Lobe/Inferior Frontal Gyrus (L) |
| DC | 0.846 | BNA | Parietal Lobe/Inferior Parietal Lobule (L) |
| WMV | 0.849 | BNA | Temporal Lobe/Fusiform Gyrus (L) |
| ALFF | 0.922 | BNA | Insular Lobe/Insular Gyrus (L) |
| WMV | 1.133 | BNA | Frontal Lobe/Orbital Gyrus (L) |
| AD | 3.857 | WMPM | Fornix (column and body of fornix) |
