# Supplementary material for: Multimodal Magnetic Resonance Imaging Reveals Aberrant Brain Age Trajectory During Youth in Schizophrenia Patients
Source: Front Aging Neurosci. 2022 Mar 3;14:823502. doi: 10.3389/fnagi.2022.823502 (PMC8929292; doi:10.3389/fnagi.2022.823502)
Supplement: Supplementary file 8 [file Table_3.DOCX]

Table S3 Performance of uncorrected BA and corrected BA in NC group

|  | r | Coefficient of determination | MAE/years | rMSE/years |
| --- | --- | --- | --- | --- |
| Uncorrected BA | 0.88 | 0.76 | 3.24 | 4.14 |
| Corrected BA | 0.90 | 0.81 | 3.08 | 3.97 |
